# Recent Studies on the Application of Microwave-Assisted Method for the Preparation of Heterogeneous Catalysts and Catalytic Hydrogenation Processes

**DOI:** 10.3390/ijms24098272

**Published:** 2023-05-05

**Authors:** Anna A. Strekalova, Anastasiya A. Shesterkina, Alexander L. Kustov, Leonid M. Kustov

**Affiliations:** 1 Laboratory of Nanochemistry and Ecology, Institute of Ecotechnologies, National University of Science and Technology MISIS, Leninsky Prospect 4, 119049 Moscow, Russia; anna.strelkova1994@mail.ru (A.A.S.); anastasiia.strelkova@mail.ru (A.A.S.); kyst@list.ru (A.L.K.); 2 Laboratory of Development and Research of Polyfunctional Catalysts, Zelinsky Institute of Organic Chemistry, Russian Academy of Sciences, Leninsky Prospekt 47, 119991 Moscow, Russia; 3 Chemistry Department, Lomonosov Moscow State University, Leninskie Gory 1/3, 119234 Moscow, Russia

**Keywords:** microwave radiation, synthesis of catalysts, selective hydrogenation

## Abstract

Currently, microwave radiation is widely used in various chemical processes in order to intensify them and carry out processes within the framework of “green” chemistry approaches. In the last 10 years, there has been a significant increase in the number of scientific publications on the application of microwaves in catalytic reactions and synthesis of nanomaterials. It is known that heterogeneous catalysts obtained under microwave activation conditions have many advantages, such as improved catalytic characteristics and stability, and the synthesis of nanomaterials is accelerated several times compared to traditional methods used to produce catalysts. The present review article is to summarize the results of modern research on the use of microwave radiation for the synthesis of heterogeneous catalytic nanomaterials and discusses the prospects for research in the field of microwave-induced liquid-phase heterogeneous catalysis in hydrogenation.

## 1. Introduction

Microwave chemistry is a step toward the future. Today, microwave heating is an integral part of industries such as food processing, pharmaceuticals, biochemistry, materials synthesis, agriculture, and also application in biomedicine or organic electronics [1,2,3,4,5]. The chemistry of materials has recently combined nanotechnology and chemistry, which has led to the search for new approaches to the synthesis of nanomaterials. In the field of synthetic chemistry, the challenge is to improve and optimize methods for obtaining materials. Replacing classical thermal heating with microwave heating is a good alternative in a wide range of chemical syntheses, especially in the cases where long-term synthetic experiments are required [6]. Over the past few years, the use of microwave irradiation has become one of the latest achievements in the field of green chemistry, as microwave heating is considered as a more efficient way to control heating in many processes, as it requires less energy than conventional methods and the microwave technology also allows the use of environmentally friendly solvents, resulting in clean products that do not require additional purification steps [7,8,9,10,11]. The use of microwave heating has expanded with the introduction of a number of environmental techniques through the use of ionic liquids and aqueous media that do not contain organic solvents and catalysts [12,13]. Modern developments in the field of microwave chemistry make it possible to selectively obtain catalytically active materials or nanomaterials as well as organic molecules with almost 100% yield and high reproducibility [14,15,16].

Currently, most research is focused on the study of microwave radiation, which is due to a number of advantages, such as uniform heating, high selectivity of processes, minimal energy consumption, and the facts that it is more environmentally friendly and more efficient compared to the processes used [17,18,19,20,21,22]. Choosing the proper catalyst for a heterogeneous catalytic process ideally suited for microwave activation is not an easy task in modern chemistry. The control of the parameters of microwave synthesis (temperature, microwave power, frequency, and pressure) and the choice of a suitable solvent opens new opportunities in the development and production of new materials.

**Figure 1:**
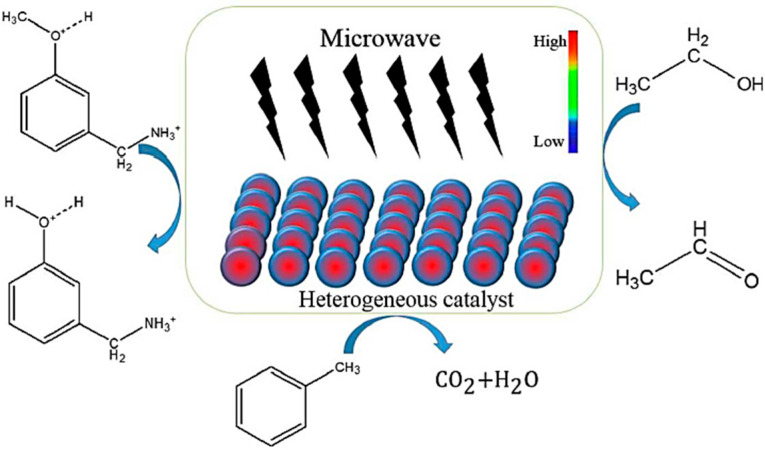
The schematic diagram of the special thermal effect of microwave on the solid catalysts (Reprinted with permission from Ref. [23]).

Recently, several review articles on the use of microwave radiation in catalysis have been published, focusing on the mechanism of the effect of microwaves on nanomaterials and a number of catalytic reactions, but the last major review was published in 2020 [23,24,25,26,27,28]. The review by Gao [23] focuses on the effect of the specific thermal effect of microwaves on liquid-phase catalytic reactions carried out on heterogeneous catalysts. It has been shown that microwave energy is absorbed by materials and converted into thermal energy, which manifests itself as a total loss of microwave energy in materials and causes local heat effects inside the catalyst grain, as shown in Figure 1. The authors report that the higher temperature of the heterogeneous catalyst caused by the microwave field can lead to higher conversion of reagents and higher reaction rates compared to traditional thermal heating.

An extensive review on microwave radiation was published by Kumar [24], which provides a comparison of microwave and traditional thermal heating, types of microwave reaction systems, solvent chemistry, and the role of microwave-assisted strategies for the synthetic chemistry. The published review exhaustively describes the advantages of microwave radiation and the possibilities and prospects of its application for various fields of science, such as solar cells technology, gas sensing, photocatalysis, batteries technology, fuel cells technology, and solvent chemistry. For this reason, in our review, we will not pay attention to these aspects from the point of view of the chemistry and physics of microwave radiation but will instead focus on the practical application of microwave exposure for the synthesis of catalytic nanomaterials, and we will also consider microwave-induced liquid-phase catalytic reactions involving hydrogen.

Our review analyzes and summarizes data on the use and influence of microwave radiation in the production of catalytic nanomaterials as well as on the use of microwave activation in heterogeneous catalytic hydrogenation reactions of various compounds.

## 2. Synthesis of Catalysts Using Microwave Radiation

Many methods of synthesis of deposited catalysts have been described in the literature, ranging from direct use of material after grinding to other methods of modification [29,30], such as co−precipitation of the carrier, co-sorption of cations, co- and sequential deposition from solutions, deposition of bimetallic colloids, and the method of deposition of components on the carrier [31]. It should be noted that there are difficulties in determining and controlling the structure of obtained bimetallic particles, plus the ratio of one metal to another one in the particle. The advantage of catalyst preparation methods based on supporting the active component onto a carrier is the effective use of the active component due to its high dispersibility. Moreover, it is necessary to carefully select sustainable and renewable sources from which the catalysts will be obtained in order to avoid harmful effects on the environment. However, a number of disadvantages should also be pointed out: restrictions on the concentration of the active component by the pore volume of the carrier, the possibility of uneven distribution of the active component across the cross section of the pellet due to the removal of the solution to the periphery of the grain during drying, etc. Thus, these synthesis methods are replaced by new synthetic approaches and methods, such as microwave synthesis, which allows the synthesis of nanomaterials in one stage, thereby reducing the synthesis time several times and accelerating the crystallization process of materials, allowing one to obtain highly dispersed nanomaterials of given sizes [32]. Research has also focused on the use of resources from waste and environmentally safe methods of catalyst synthesis [33].

**Figure 2:**
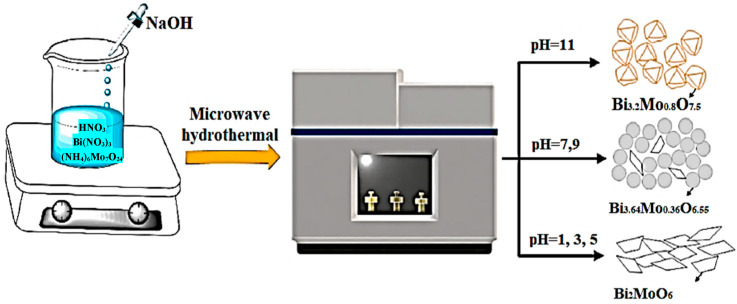
The schematic illustration of the formation of bismuth molybdate catalysts (Reprinted with permission from Ref. [35]).

The hydrothermal method [34] applied under microwave radiation is used to regulate the morphology of materials to obtain excellent physical and chemical properties. Recently, it has been shown that this method is well suited for the synthesis of molybdates [30]. In this work, six different bismuth molybdate-based catalysts were investigated and synthesized by a fast hydrothermal method using microwave irradiation under different pH conditions. By adjusting the pH value during preparation, the morphology and structure of the synthesized catalysts can be modified as well as the transition of Bi_2_MoO_6_ crystals to Bi_3.2_Mo_0.8_O_7.5_, as shown in Figure 2. Crystals can be obtained selectively. The sample prepared at pH 1 showed excellent activity for the oxidation of sulfur compounds in liquid fuel at 60 °C with a hydrogen peroxide to sulfur molar ratio of four, achieving the removal of dibenzothiophene, 4,6−dimethyldibenzothiophene, and benzothiophene with an efficiency reaching 99.71%, 99.68%, and 77.95%, respectively [35].

**Figure 3:**
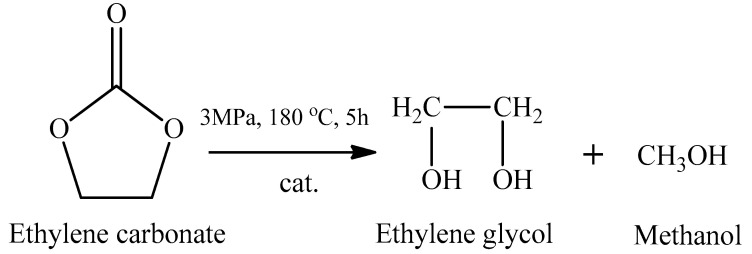
The schematic reaction of hydrogenolysis of ethylene carbonate.

Composite catalysts Cu−CeO_2_/C with a developed porous structure for selective hydrogenolysis of ethylene carbonate were prepared by the carbonization−impregnation method using microwave radiation, and, for comparison, similar catalysts were synthesized by the impregnation method. This reaction drew attention to the environmentally safe synthesis of sustainable chemical raw materials and fuels. The morphology of the Cu-CeO_2_/C catalyst series was revealed using physicochemical methods of analysis, and it was shown that Cu−CeO_2_/C catalysts with corresponding copper−cerium interactions improve the dispersion of copper particles and provide a higher Cu^+^/(Cu^+^ + Cu^0^) ratio and a higher concentration of oxygen vacancies at the surface. These interactions result in enhanced adsorption of ethylene carbonate and high hydrogenation activity. The reaction was carried out in an autoclave, at 3 MPa H_2_, 180 °C, for 5 h, as shown in Figure 3. The developed Cu−CeO_2_/C catalyst showed a higher catalytic activity (the conversion reached 92%) in hydrogenation of ethylene carbonate compared to the impregnated Cu−CeO_2_/C catalyst (the conversion was about 60%), which was explained by the interaction effect created by CeO_2_ doping. At the same time, the authors proposed a methodology for the synthesis of a porous metal−metal oxide catalyst on a carbon carrier for heterogeneous hydrogenation [36].

A new microwave synthesis of copper phyllosilicates on a commercial SiO_2_ carrier was first developed [37]. This technique allows a significant reduction of the sample synthesis time from 9 h to 6 h relative to the method of thermal decomposition of urea. The samples were synthesized in a Multiwave Pro microwave unit under irradiation (2.45 GHz) with urea in four Teflon autoclave-type vessels for 6 h. The morphology of the obtained catalysts was studied by XRD, TEM, and N_2_ adsorption techniques, and the formation of chrysocolla phases in the samples was confirmed. The catalysts prepared by the microwave method were highly efficient in the selective hydrogenation of the C≡C bond in 1,4−butynediol to 1,4−butenediol and 2−phenylacetylene, with the selectivity of 96.5% and 100% at the complete conversion for 2 and 0.5 h of the reaction, respectively. The resulting method of microwave synthesis showed enough advantages to be considered the most preferable alternative to the traditional methods.

Akay et al. [38,39,40] considered applied heterogeneous Fe, Co, and Ni catalysts as primary precursors and Ca, Mn, and Cu as precursors for binary SiO_2_ based catalytic systems, induced by microwave (as well as solar) radiation from the liquid state, and represent new catalysts with such properties as high porosity, surface area, chemical and morphological heterogeneity, oxygen, and cation vacancies. These catalysts have been successfully used to provide extremely high conversions––for example, in ammonia synthesis compared to existing catalysts. The performance of the catalyst was inferior when using thermal methods compared to microwave irradiation. In addition, the use of the classic incipient wetting impregnation method also resulted in poorer catalyst performance.

**Figure 4:**
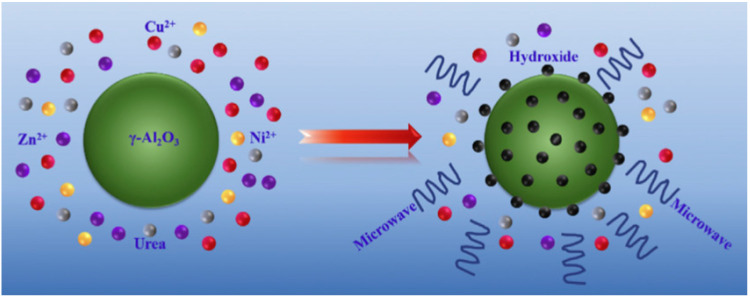
The catalyst generation process (Reprinted with permission from Ref. [41]).

Jing et al. [41] produced a copper−based catalyst by thermal hydrolysis of urea using microwave radiation to produce hydrogen from methanol decomposition, as shown in Figure 4. The synthesis of the catalysts was performed in such a way that when the temperature of the solution heated uniformly by microwaves reached 80 °C, the hydroxyl ion could be formed during the decomposition of urea, which could form hydroxides with Cu^2+^, Ni^2+^, and Zn^2+^ on the surface of the carrier γ-Al_2_O_3_. It was found that the content of Cu gradually decreased with increasing microwave heating temperature, while the content of Ni and Zn increased with increasing microwave heating temperature, as shown in Table 1. This result is explained by the difference in solubility of metal hydroxides and solution pH. The characterization results show that there is a well-defined correlation between the catalytic characteristics of the catalysts and the microwave heating temperature. 

The catalysts synthesized on the basis of Pd on γ-Al_2_O_3_, which was developed by a simple and environmentally friendly microwave synthesis, deserve attention. As a model reaction, this catalyst was tested in the hydrogenation of cinnamic aldehyde to hydrocinnamic aldehyde using very “mild” reaction conditions (2 MPa, 100 °C, 3 h) and short irradiation times, without the use of any polymeric stabilizer. This reaction is of significant industrial and pharmacological interest; in fact, hydrocinnamic aldehyde may be an important intermediate product for the production of pharmaceuticals used to treat HIV [42]. The obtained Pd/γ-Al_2_O_3_ nanocatalysts showed interesting catalytic characteristics in terms of both activity and selectivity, achieving the complete substrate conversion and selectivity up to 97% for hydrocinnamic aldehyde [43].

Nishida et al. [44] reported a method for a simple and rapid production of size-controlled Rh nanoparticles by microwave chemical reduction using alcohol. The alcohol acts as a reducing agent and solvent, and it is very efficient for changing the size of Rh particles. Using ethanol, which exhibits reducing properties, small Rh particles with high catalytic activity for CO oxidation were obtained. In addition, a Rh catalyst with a Rh particle size of 2.7 nm showed high activity in the hydrogenation of benzonitrile to a secondary imine and showed reusability in the hydrogenation of nitriles (30 °C, 3 bar H_2_) [45]. Ru, Rh, Pd, Ir, and Pt nanoparticles stabilized by poly(N-vinyl-2-pyrrolidone) (PVP) with a uniform size were prepared by chemical reduction with ethanol in the microwave oven [46]. All metallic nanoparticles had a similar size and the same amount of PVP. The catalytic efficiency of the prepared metal nanoparticles was evaluated for the hydrogenation of benzonitrile under ambient conditions (25 °C, 1 bar H_2_). Rh nanoparticles showed the highest benzonitrile conversion and the highest selectivity for the secondary imine product.

Lingaya et al. [47] synthesized a series of SiO_2_−based Pd-Fe catalysts by incipient wetness impregnation of the carrier with Fe and Pd nitrate precursors, the catalysts were dried at 120 °C for 2 h, one part was calcined in an air atmosphere at 450 °C for 5 h, and the remaining part was microwave irradiated (irradiation at 100% power for 5 min). The catalysts prepared by the microwave irradiation method showed higher hydrodechlorination activity compared to the impregnation method. The addition of Fe to Pd reduced the activity, by diluting Pd or forming a Pd−Fe alloy. Microwave irradiation increases the palladium particle size and reduces alloy formation while maintaining the activity. Silica gel provides a clear indication of the change in the morphology of the active particles.

The synthesized bimetallic particles of ruthenium−palladium (Ru−Pd) and ruthenium−nickel (Ru−Ni) nanoalloys with different metal compositions were prepared by solvothermal treatment with microwave irradiation using PVP as a capping agent and ethylene glycol as a solvent and a reducing agent. The synthesized bimetallic nano−alloy particles were then deposited on γ-Al_2_O_3_ to obtain supported nano-alloy catalysts. The hydrogenation of dibenzo−18−crown−6 ester (DB18C6) was carried out at 9 MPa, 120°C and 3.5 h using the synthesized bimetallic nanoalloy catalysts. It was observed that the bimetallic nanocatalyst synthesized by the microwave method at Ru:Pd 3:1% (wt.) exhibited a higher catalytic activity and resulted in a 98.9% conversion of DB18C6 with a 100% selectivity towards cis−cis dicyclohexano−18−crown−6 ester (CSC DCH18C6), showing better results compared to 4 wt% Ru/γ-Al_2_O_3_ microwave irradiated (MWI) and 5 wt.% Ru/γ-Al_2_O_3_ conventionally treated nanocatalysts [48].

The effect of the catalyst carrier, reaction medium, pressure, temperature, and initial concentration of levulinic acid (LA) was investigated [49] to obtain optimal conditions for high yields of γ−valerolactone (GVL). For this purpose, Li et al. proposed the synthesis of a Ru-based catalyst, which was prepared by a one-step microwave thermolytic process using dodecacarbonyltriruthenium [Ru_3_(CO)_12_] as a precursor. Generally, the support and Ru_3_(CO)_12_ were placed in an agate mortar and grinded for 20 min. The precursor mixture was then placed in a reactor with a quartz tube with an inner diameter of about 10 mm and purged with argon for 2 h at room temperature to remove oxygen in the reactor, and the reaction was performed in an inert atmosphere. The reactor was then placed in a household microwave oven operating at 2.45 GHz and 800 W. Finally, the obtained products were cooled to room temperature under argon. Activated coconut shell carbon, carbon nanotubes, functionalized carbon nanotubes, and γ-Al_2_O_3_ were used as carriers. For comparison, a sample was synthesized by incipient wet impregnation of γ-Al_2_O_3_ with ruthenium chloride solution in a sufficient concentration to obtain a solid containing 5% Ru. The sample was vacuum dried at 100 °C for 10 h. GVL was obtained in a high yield (99%) by aqueous−phase hydrogenation of LA in the presence of supported Ru catalysts. The catalyst prepared by the microwave thermolytic method shows the best catalytic performance compared to other systems, at 100 °C, 2.0 MPa. This is due to the high dispersion of Ru particles on the active carbon substrate.

## 3. Microwave−Assisted Catalytic Hydrogenation

### 3.1. Hydrogenation of Aldehydes to Alcohols

The vast majority of reactions proceed at elevated temperatures, provided that the process conditions (temperature, MW radiation power, time, solvent, catalyst, ratios, and amounts of reagents) are chosen correctly, thereby proceeding faster and with higher yields. The hydrogenation process is one of the most important and widespread processes in industry, as it makes it possible to obtain a huge range of valuable organic compounds. Selective hydrogenation of carbonyl compounds is of great industrial and scientific interest due to wide application of unsaturated alcohols in pharmaceutical and food industries; in chemical production as intermediates for synthetic polymers, plasticizers, and solvents; and in fine organic synthesis [50]. An additional advantage of the process is the possibility of obtaining aldehydes and ketones from bioavailable materials. Difficulties encountered by researchers in the hydrogenation of carbonyl substrates include the presence of a conjugated C=C bond in aldehydes such as cinnamaldehyde and citral, the presence of an aromatic ring as in benzaldehyde, or steric hindrances in various ketones. One of the most common methods for hydrogenation of carbonyl compounds involves the use of stoichiometric reducing agents such as sodium borohydride and hydrazine hydrate. The obvious disadvantages of this technique are toxicity, explosiveness of the reducing agents, and the large amount of waste produced during the reaction. The use of homogeneous catalysts is complicated by the necessity of their separation from the reaction mixture and the quite severe conditions that are usually required. The use of heterogeneous catalysts is becoming increasingly popular, as this approach is consistent with the principles of “green chemistry”. It also makes it easy to separate the catalyst from the reaction mixture and to recycle it. However, until now, heterogeneous hydrogenation proceeds at higher temperature and pressure, which makes the process non-selective, expensive, and requires additional resources.

**Figure 5:**
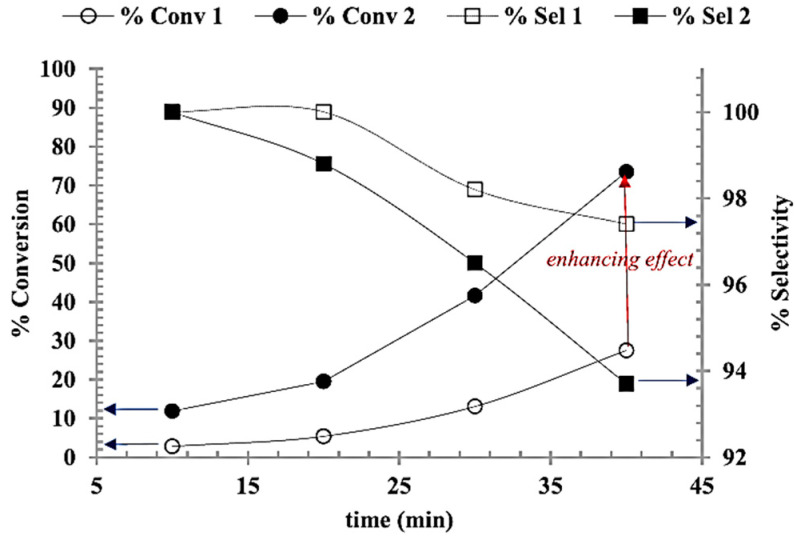
Hydrogenation of CAL ○ % Conv 1: conventional heating, •% Conv 2: microwave irradiation, □ % Sel 1: conventional heating and ■ Sel 2: microwave irradiation (Reprinted with permission from Ref. [51]).

In recent years, Iqbal et al. [51] have actively studied the catalytic activity of catalysts based on palladium/zirconia in the hydrogenation of cinnamyl aldehyde to cinnamyl alcohol, both in microwave conditions and in an autoclave. The reaction conditions differed slightly: a CEM unit at 1.034 MPa H_2_, 120 °C, 40 min was used in microwave hydrogenation, and a Parr autoclave applied under the thermal control also included stirring at the speed of 1000 rpm. Thus, a comparison of thermal and microwave hydrogenation under optimized reaction conditions was made. The conversion percentage with microwave irradiation is much higher compared to the conventional heating system. The conversion for both systems increase linearly with time, and the maximum conversions observed for the microwave and pressurized reactor were 73.5% and 27%, respectively, as shown in Figure 5. Thus, the microwave hydrogenation system demonstrates improved performance compared to the conventional pressurized autoclave system.

In the model reaction of hydrogenation of octanal to octanol by molecular hydrogen, microwave irradiation enhances the catalytic activity of tetragonal ZrO_2_ [52]. The reaction was carried out under optimal reaction parameters such as microwave power, a temperature of 110 °C, a catalyst (t−ZrO_2_), P_H2_ 1 atm, and a reaction time of 1 h 20 min in a solvent-free system. The selectivity to octanol as a target product at a 22% conversion was 99% in the microwave system, which is 1.5 times higher than the value obtained in the traditional heating method. Therefore, the microwave heating is more efficient and more reproducible results are obtained than the conventional heating system due to the safe, environmentally friendly, less labor-intensive, and economical procedure for catalytic conversion of octanal to octanol.

**Figure 6:**
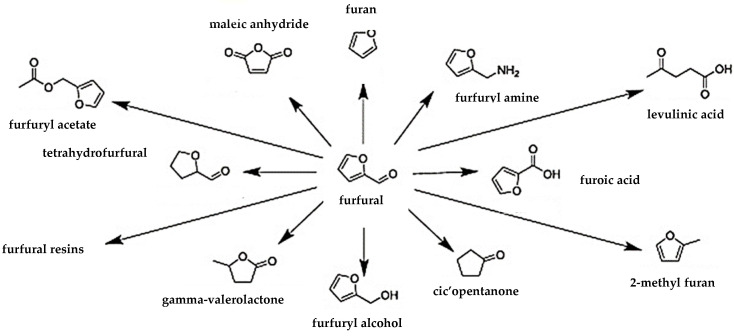
Furfural derivatives (Reprinted with permission from Ref. [53]).

One of the interesting platform chemicals derived from biomass, due to its availability, is furfural. It is used in agrochemistry, perfumery, and plastic production, but it is also applied as a substrate for transformation into value-added products by hydrogenation, oxidation, decarboxylation, and condensation reactions to form furfural derivatives, as shown in Figure 6 [53].

Traditionally, the hydrogenation reaction of furfural to furfuryl alcohol was carried out both in the gas and liquid phase, because the evaporation of furfural requires significant energy and high temperatures (in the gas phase), while in the liquid phase, a high hydrogen pressure is required, which increases the cost of the product [54]. In most cases, good results can be achieved with the use of noble metals, which increases material costs, toxicity, and requires severe reaction conditions.

Ronda-Leal et al. [53] showed that hydroconversion of furfural was first studied using TiO_2_−Fe_2_O_3_/C as a catalyst. The solvent in all experiments was a mixture of isopropanol and formic acid, which was the main hydrogen donor in the chemical reaction. The reaction was carried out in a CEM Discover 2.0 microwave reactor at 200 °C for 15 min and with continuous flow. The selectivity of the formation of the target product at a 70% conversion was 100%, thus representing significant progress in the development of strategies for selective biomass conversion given the uncontrollable reactivity of such molecules, which leads to a number of reaction byproducts.

### 3.2. Selective Reduction of Nitrobenzene to Aniline

The reduction of aromatic nitro compounds is an important transformation that has been widely studied because anilines are used in the synthesis of pharmaceuticals and agrochemicals. Selective and complete reduction of nitrobenzene in the presence of glycerol used as a hydrogen source has been performed using Raney nickel [55] and in the presence of a recyclable catalyst based on magnetic ferrite−nickel nanoparticles. Despite the disadvantage of high viscosity at room temperature, glycerol is an optimal solvent for catalysis purposes because of its high polarity and ability to remain in the liquid phase over a large temperature range (from 17.8 to 290 °C). It also has a low vapor pressure, which means that it can be used under microwave irradiation conditions. Thus, copper nanoparticles (CuNP) were prepared in glycerol [56], and the efficiency of the glycerol layer interaction with the metal active centers was investigated by HRTEM analysis. Its high polarity, low vapor pressure, long relaxation time, and high acoustic impedance meant that excellent results were also obtained when the reaction medium was subjected to ultrasonic irradiation. Microwave synthesis was shown to play an important role in this process due to its ability to improve CuNP dispersion, promote mechanical depassivation, and increase the catalytically active surface, while MW irradiation reduces the reaction time from hours to minutes. These synergistic combinations contributed to the exhaustive reduction of nitrobenzene to aniline and facilitated the expansion of the protocol for its optimized use in industrial MW reactors.

### 3.3. Selective Hydrogenation of Levulinic Acid

Levulinic acid and its derivatives are promising platform chemicals that can be obtained from biomass. It can be converted into value-added molecules that can be used as environmentally friendly solvents and additives to biofuels in the pharmaceutical industry and in the synthesis of biopolymers [57]. Currently, most of the research is focused on the hydrogenation of levulinic acid into γ−valerolactone. γ−Valerolactone is a valuable chemical compound, a platform molecule, considered as an intermediate for the synthesis of value-added chemical compounds, components of motor fuels, and biopolymers. This substance is well established as an environmentally friendly solvent, fuel additive, flavoring agent, and food additive [58]. A study was performed on the hydrogenation of levulinic acid using microwave synthesis on gold catalysts (commercial 1 wt% Au/TiO_2_ using AUROlite™ (catalogue number 79-0165, CAS number 7440-57-5, Strem Chemicals INC) and 2.5 wt% Au/ZrO_2_ prepared by precipitation−sedimentation) to produce 1,4−pentanediol (1,4−PDO) [59]. Hydrogenation of levulinic acid under microwave conditions was tested in a SynthWAVE reactor with a closed microwave cavity. The hydrogenation was carried out both in the absence of a solvent and in the presence of H_2_O used as a solvent. The mixture was heated under MW and magnetic stirring for 4 h at 150 °C. Interestingly, the selectivity to 1,4−PDO was close to 100% at 200 °C. The extended characterization highlighted the joint role played by the gold nanoparticles and the support on which the activated hydrogen atoms are spilled over to react with LA. This results in remarkable Au/TiO_2_ activity. Both catalysts showed structural and morphological stability under reaction conditions.

**Figure 7:**
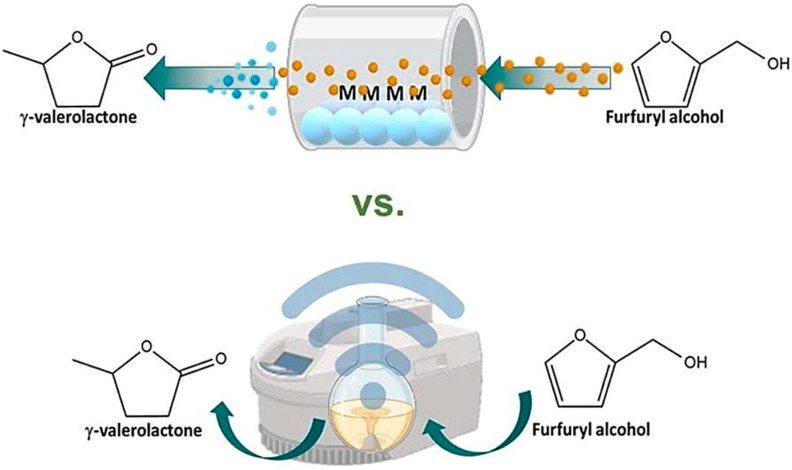
One−pot multi-step synthesis of γ−valerolactone from furfuryl alcohol (Reprinted with permission from Ref. [60]).

The catalytic activity of the synthesized Al−SBA−15 mesoporous materials was evaluated in the single−reactor conversion of furfuryl alcohol (FAL) using 2−propanol as an H−donor solvent to yield γ−valerolactone (GVL) under both microwave irradiation (microwave synthesizer Discover^®^ 2.0, CEM Corporation’s, US) and continuous flow conditions (Phoenix Flow Reactor^TM^, ThalesNano, Budapest), as shown in Figure 7. All materials exhibited conversions up to 99% with GVL selectivities of ca. 20−41% after one hour of the reaction. A study of the recyclability of the materials showed good GVL production over three reaction cycles using both microwave and flow reaction conditions [60].

### 3.4. Selective Catalytic Transfer Hydrogenation

Catalytic transfer hydrogenation (CTH) is a new alternative process for selective hydrogenation, and the microwave irradiation is an efficient heating method for the initiation of the organic reaction.

CTH of Jatropha oil biodiesel was performed [61] by microwave heating using Raney nickel as a catalyst and water as a solvent, as well as by conventional heating. The effect of operating parameters on the composition of the upgraded biodiesel was analyzed in detail and optimal conditions for CTH of Jatropha oil biodiesel were found. Under optimal conditions, the mass conversion of methyl linoleate reached 91.98 wt.% with the reaction time of 50 min. For the improved biodiesel obtained after 50 min, the methyl linoleate content, methyl oleate content, methyl stearate content and iodine number were 2.45 wt.%, 76.70 wt.%, 8.45 wt.% and 70.21 wt.%, respectively. Thus, the use of microwave heating in the CTH reaction can shorten the hydrogenation time and speed up the hydrogenation process, which helps to reduce the energy consumption for the reaction.

The CTH of polyunsaturated fatty acid methyl esters was carried out [62] by using a Pd/organobentonite catalyst with water as a solvent and ammonium formate as a hydrogen donor under microwave heating. The effects of CTH reaction conditions, including the amount of ammonium formate, the amount of the solvent, the dosage of the catalyst, reaction temperature, reaction time, and agitation rate, on the hydrogenation process was studied systematically. Meanwhile, the effects of both microwave heating and conventional heating on the CTH reaction have been studied. Under the optimal CTH conditions (40 g as the amount of ammonium formate, 60 g as the water amount, 8 wt.% as the catalyst dosage, 80 °C as the reaction temperature, 140 min as the reaction time, and 350 rpm as the agitation speed), methyl linoleate was successfully hydrogenated into methyl oleate with a high conversion ratio of 78.56%, high methyl oleate yield of 72.22%, and high selectivity for the cis−isomer of methyl oleate of 70.29%. Compared with conventional heating, microwave heating used in the CTH process could enhance the conversion from 60.27% to 78.56% and reduce the hydrogenation time.

## 4. Conclusions

Considering the great interest of the scientific community over the past 10 years in the application of microwave radiation in various catalytic processes and for the synthesis of nanomaterials and nanotechnologies, we can confidently say that catalysis is at the forefront of microwave research. A huge number of scientific articles, books, and reviews have been devoted to the significant advantages of using microwave radiation, but all known methodologies are used exclusively in lab-scale fundamental research. It is well known that the interaction between microwave irradiation and a heterogeneous catalyst can lead to a local thermal effect inside the catalyst grain, which contributes to the intensification of the catalytic reaction and increases the efficiency of the process. However, the problem of scaling up catalytic processes using microwave radiation to large-scale industrial production has not yet been solved, since this requires an increase in the size of the catalytic equipment and the amount of a catalyst, which will lead to a change in the frequency of microwaves by tens of times and will probably lead to a decrease in the reachable temperature and a drop in efficiency. We expect that the mechanism of the effect of microwave radiation in solutions and on heterogeneous nanomaterials will be actively investigated to improve understanding of the processes that are occurring in heterogeneous catalytic reactions. We believe that the interconnection of rapidly developing modern science and engineering developments will allow one to create more environmentally friendly and efficient catalytic nanomaterials in compliance with the principles of “green” chemistry.

## Figures and Tables

**Table 1:** Elemental composition and textural properties of catalysts and γ-Al_2_ O_3_ carrier, crystal size of Cu in reduced catalysts (Adapted with permission from [41]).

**Sample**	**Cu (wt%) ^a^**	**Ni** **(wt%)**	**Zn** **(wt%)**	**Cu Crystallite Size (nm) ^b^**	**S_BET_** **(m^2^g^−1^)**	**V** **(cm^3^g^−1^)**	**D_BJH_** **(nm)**
γ-Al_2_O_3_	-	-	-	-	164.1	0.3842	9.37
MW-Cu/Ni-80	47.92	0.21	6.23	22	71.9	0.1670	9.29
MW-Cu/Ni-85	35.33	1.61	31.83	16	56.7	0.1689	11.92
MW-Cu/Ni-90	28.03	3.00	35.30	14	57.9	0.1770	12.22
MW-Cu/Ni-95	27.32	3.40	42.60	10	79.8	0.2522	12.64

^a^ Determined by ICP-OES. ^b^ Calculated by Cu (111) (43,29^0^)from the Scherrer formula.

## Data Availability

Data are contained within the article.

## References

[1] Tsuji M. (2017). Microwave−Assisted Synthesis of Metallic Nanomaterials in Liquid Phase. Chem. Select..

[2] Torres-Moya I., Harbuzaru A., Donoso B., Prieto P., Ponce Ortiz R., Díaz-Ortiz Á. (2022). Microwave Irradiation as a Powerful Tool for the Preparation of n-Type Benzotriazole Semiconductors with Applications in Organic Field-Effect Transistors. Molecules.

[3] Chen Z., Wu Q., Guo W., Niu M., Tan L., Wen N., Zhao L., Fu C., Yu J., Ren X. (2021). Nanoengineered biomimetic Cu-based nanoparticles for multifunational and efficient tumor treatment. Biomaterials.

[4] Liang K.-H., Som S., Gupta K.K., Lu C.-H. (2022). Electrochemical characterization of TiNb_2_O_7_ as anode material synthesized using microwave-assisted microemulsion route. J. Am. Ceram. Soc..

[5] Henam S.D., Ahmad F., Shan M.A., Parveen S., Wani A.H. (2019). Microwave synthesis of nanoparticles and their antifungal activities. Spectrochim. Acta A Mol. Biomol. Spectrosc..

[6] Jiang S., Daly H., Xiang H., Yan Y., Zhang H., Hardacre C., Fan X. (2019). Microwave-assisted catalyst-free hydrolysis of fibrous cellulose for deriving sugars and biochemicals. Front. Chem. Sci. Eng..

[7] Horikoshi S., Arai Y., Ahmad I., DeCamillis C., Hicks K., Schauer B., Serpone N. (2020). Application of Variable Frequency Microwaves in Microwave-Assisted Chemistry: Relevance and Suppression of Arc Discharges on Conductive Catalysts. Catalysts.

[8] Tompsett G.A., Conner W.C., Yngvesson K.S. (2006). Microwave Synthesis of Nanoporous Materials. Chem. Phys. Chem..

[9] Xie X., Zhou Y., Huang K. (2019). Advances in Microwave-Assisted Production of Reduced Graphene Oxide. Front. Chem..

[10] Kostyukhin E.M., Kustov A.L., Evdokimenko N.V., Bazlov A.I., Kustov L.M. (2020). Hydrothermal microwave-assisted synthesis of LaFeO_3_ catalyst for N_2_O decomposition. J. Am. Ceram. Soc..

[11] Jin J., Wen Z., Long J., Wang Y., Matsuura T., Meng J. (2000). One-Pot Diazo Coupling Reaction Under Microwave Irradiation in the Absence of Solvent. Synth. Commun..

[12] George N., Singh G., Singh R., Singh G., Devi A., Singh H., Kaur G., Singh J. (2022). Microwave accelerated green approach for tailored 1,2,3–triazoles via CuAAC. Sustain. Chem. Pharm..

[13] Zamri A.A., Ong M.Y., Nomanbhay S., Show P.L. (2021). Microwave plasma technology for sustainable energy production and the electromagnetic interaction within the plasma system: A review. Int. J. Environ. Res..

[14] Kustov L.M., Kustov A.L., Salmi T. (2022). Microwave-Assisted Conversion of Carbohydrates. Molecules.

[15] Palanisamy S., Wang Y.-M. (2019). Superparamagnetic iron oxide nanoparticulate system: Synthesis, targeting, drug delivery and therapy in cancer. Dalton Trans..

[16] Kustov L.M., Kustov A.L., Salmi T. (2022). Processing of lignocellulosic polymer wastes using microwave irradiation. Mendeleev Commun..

[17] Gao X., Shu D., Li X., Li H. (2019). Improved film evaporator for mechanistic understanding of microwave-induced separation process. Front. Chem. Sci. Eng..

[18] Li H., Zhao Z., Xiouras C., Stefanidis G.D., Li X., Gao X. (2019). Fundamentals and applications of microwave heating to chemicals separation processes. Renew. Sust. Energ. Rev..

[19] Kostyukhin E.M., Kustov A.L., Kustov L.M. (2019). One-step hydrothermal microwave-assisted synthesis of LaFeO_3_ nanoparticles. Ceram. Int..

[20] Vakili R., Xu S., Al-Janabi N., Gorgojo P., Holmes S.M., Fan X. (2018). Microwave-assisted synthesis of zirconium-based metal organic frameworks (MOFs): Optimization and gas adsorption. Microporous Mesoporous Mater..

[21] Chia S.R., Nomanbhay S., Milano J., Chew K.W., Tan C.-H., Khoo K.S. (2022). Microwave-Absorbing Catalysts in Catalytic Reactions of Biofuel Production. Energies.

[22] Kostyukhin E.M. (2018). Synthesis of Magnetite Nanoparticles upon Microwave and Convection Heating. Russ. J. Phys. Chem. A.

[23] Li H., Zhang C., Pang C., Li X., Gao X. (2020). The Advances in the Special Microwave Effects of the Heterogeneous Catalytic Reactions. Front. Chem..

[24] Kumar A., Kuang Y., Liang Z., Sun X. (2020). Microwave Chemistry, Recent Advancements and Eco-Friendly Microwave-Assisted Synthesis of Nanoarchitectures and Their Applications: A Review. Mater. Today Nano.

[25] Kostyukhin E.M., Kustov L.M. (2018). Microwave-assisted synthesis of magnetite nanoparticles possessing superior magnetic properties. Mendeleev Commun..

[26] Verma C., Quraishi M.A., Ebenso E.E. (2018). Microwave and ultrasound irradiations for the synthesis of environmentally sustainable corrosion inhibitors: An overview. Sustain. Chem. Pharm..

[27] Budarin V.L., Shuttleworth P.S., De Bruyn M., Farmer T.J., Gronnow M.J., Pfaltzgraff L., Macquarrie D.J., Clark J.H. (2015). The potential of microwave technology for the recovery, synthesis and manufacturing of chemicals from bio-wastes. Catal. Today.

[28] El Khaled D., Novas N., Gazquez J.A., Manzano-Agugliaro F. (2018). Microwave dielectric heating: Applications on metals processing. Renew. Sust. Energ. Rev..

[29] Haruta M. (2003). When Gold Is Not Noble: Catalysis by Nanoparticles. Chem. Rec..

[30] Kostyukhin E.M., Nissenbaum V.D., Abkhalimov E.V., Kustov A.L., Ershov B.G., Kustov L.M. (2020). Microwave-Assisted Synthesis of Water-Dispersible Humate-Coated Magnetite Nanoparticles: Relation of Coating Process Parameters to the Properties of Nanoparticles. Nanomaterials.

[31] Zhang Y. (2020). Preparation of heterogeneous catalysts based on CWAO technology. J. Phys. Conf. Ser..

[32] Schutz M.B., Xiao L., Lehnen T., Fischer T., Mathur S. (2017). Microwave-assisted synthesis of nanocrystalline binary and ternary metal oxides. Int. Mater. Rev..

[33] Khan H.M., Iqbal T., Mujtaba M.A., Soudagar M.E.M., Veza I., Fattah I.M.R. (2021). Microwave Assisted Biodiesel Production Using Heterogeneous Catalysts. Energies.

[34] Hare D.O. (2001). Hydrothermal Method. Encyclopedia of Materials: Science and Technology.

[35] Zhang Z., Zeng X., Wen L., Liao S., Wu S., Zeng Y., Zhou R., Shan S. (2023). Microwave-assisted rapid synthesis of bismuth molybdate with enhanced oxidative desulfurization activity. Fuel.

[36] Song W., Cai W., Hu S., Jiang X., Lai W. (2022). Synergistic effect between CeO_2_ and Cu for ethylene carbonate hydrogenation. J. Porous Mater..

[37] Shesterkina A., Vikanova K., Kostyukhin E., Strekalova A., Shuvalova E., Kapustin G., Salmi T. (2022). Microwave Synthesis of Copper Phyllosilicates as Effective Catalysts for Hydrogenation of C≡C Bonds. Molecules.

[38] Akay G. (2016). Co-Assembled Supported Catalysts: Synthesis of Nano-Structured Supported Catalysts with Hierarchic Pores through Combined Flow and Radiation Induced Co-Assembled Nano-Reactors. Catalysts.

[39] Akay G. (2020). Plasma Generating—Chemical Looping Catalyst Synthesis by Microwave Plasma Shock for Nitrogen Fixation from Air and Hydrogen Production from Water for Agriculture and Energy Technologies in Global Warming Prevention. Catalysts.

[40] Akay G. (2017). Sustainable Ammonia and Advanced Symbiotic Fertilizer Production Using Catalytic Multi-Reaction-Zone Reactors with Nonthermal Plasma and Simultaneous Reactive Separation. ACS Sustain. Chem. Eng..

[41] Jing J., Li L., Chu W., Wei Y., Jiang C. (2018). Microwave-assisted synthesis of high performance copper-based catalysts for hydrogen production from methanol decomposition. Int. J. Hydrogen Energy.

[42] Muller A., Bowers J. (1997). Processes for Preparing Hydrocinnamic Acid. WO Patent.

[43] Galletti AM R., Antonetti C., Venezia A.M., Giambastiani G. (2010). An easy microwave-assisted process for the synthesis of nanostructured palladium catalysts and their use in the selective hydrogenation of cinnamaldehyde. Appl. Catal. A Gen..

[44] Nishida Y., Sato K., Yamamoto T., Wu D., Kusada K., Kobayashi H., Matsumura S., Kitagawa H., Nagaoka K. (2017). Facile Synthesis of Size-controlled Rh Nanoparticles via Microwave-assisted Alcohol Reduction and Their Catalysis of CO Oxidation. Chem. Lett..

[45] Nishida Y., Chaudhari C., Imatome H., Sato K., Nagaoka K. (2017). Selective Hydrogenation of Nitriles to Secondary Imines over Rh-PVP Catalyst under Mild Conditions. Chem. Lett..

[46] Nishida Y., Wada Y., Chaudhari C., Sato K., Nagaoka K. (2019). Preparation of Noble-metal Nanoparticles by Microwave-assisted Chemical Reduction and Evaluation as Catalysts for Nitrile Hydrogenation under Ambient Conditions. J. Jpn. Pet. Inst..

[47] Lingaiah N., Sai Prasad P., Kanta Rao P., Berry F., Smart L. (2002). Structure and activity of microwave irradiated silica supported Pd–Fe bimetallic catalysts in the hydrodechlorination of chlorobenzene. Catal. Commun..

[48] Suryawanshi Y.R., Chakraborty M., Jauhari S., Mukhopadhyay S., Shenoy K.T. (2019). Hydrogenation of Dibenzo-18-Crown-6 Ether Using γ-Al_2_O_3_ Supported Ru-Pd and Ru-Ni Bimetallic Nanoalloy Catalysts. Int. J. Chem. React. Eng..

[49] Li C., Ni X., Di X., Liang C. (2018). Aqueous phase hydrogenation of levulinic acid to γ-valerolactone on supported Ru catalysts prepared by microwave-assisted thermolytic method. J. Fuel Chem. Technol..

[50] Nongwe I., Ravat V., Meijboom R., Coville N.J. (2016). Pt supported nitrogen doped hollow carbon spheres for the catalysed reduction of cinnamaldehyde. Appl. Catal. A Gen..

[51] Iqbal Z., Sadiq M., Sadiq S., Saeed K. (2021). Selective hydrogenation of cinnamaldehyde to cinnamyl alcohol over palladium/zirconia in microwave protocol. Catal. Today.

[52] Iqbal Z., Sadiq S., Sadiq M., Khan I., Saeed K. (2021). Effect of Microwave Irradiation on the Catalytic Activity of Tetragonal Zirconia: Selective Hydrogenation of Aldehyde. Arab. J. Sci. Eng..

[53] Ronda-Leal M., Osman S.M., Jang H.W., Shokouhimehr M., Romero A.A., Luque R. (2023). Selective hydrogenation of furfural using TiO_2_-Fe_2_O_3_/C from Ti-Fe-MOFs as sacrificial template: Microwave vs Continuous flow experiments. Fuel.

[54] Wang X., Rinaldi R. (2012). Exploiting H-transfer reactions with RANEY^®^ Ni for upgrade of phenolic and aromatic biorefinery feeds under unusual, low-severity conditions. Energy Environ. Sci..

[55] Wolfson A., Dlugy C., Shotland Y., Tavor D. (2009). Glycerol as solvent and hydrogen donor in transfer hydrogenation–dehydrogenation reactions. Tetrahedron Lett..

[56] Moran M.J., Martina K., Stefanidis G.D., Jordens J., Gerven T.V., Goovaerts V., Manzoli M., Groffils C., Cravotto G. (2020). Glycerol: An Optimal Hydrogen Source for Microwave-Promoted Cu-Catalyzed Transfer Hydrogenation of Nitrobenzene to Aniline. Front. Chem..

[57] Rackemann D.W., Doherty W.O. (2011). The conversion of lignocellulosics to levulinic acid. Biofuels Bioprod. Biorefining.

[58] Taran O.P., Sychev V.V., Kuznetsov B.N. (2021). γ-Valerolactone as a promising solvent and basic chemical product. Catalytic synthesis from components of vegetable biomass. Catal. Prom..

[59] Bucciol F., Tabasso S., Grillo G., Menegazzo F., Signoretto M., Manzoli M., Cravotto G. (2019). Boosting levulinic acid hydrogenation to value-added 1,4-pentanediol using microwave-assisted gold catalysis. J. Catal..

[60] Lazaro N., Ronda-Leal M., Pineda A., Osman S.M., Shokouhimehr M., Jang H.W., Luque R. (2023). One-pot multi-step synthesis of gamma-valerolactone from furfuryl alcohol: Microwave vs continuous flow reaction studies. Fuel.

[61] Wei G., Liu Z., Zhang L., Li Z. (2018). Catalytic upgrading of Jatropha oil biodiesel by partial hydrogenation using Raney-Ni as catalyst under microwave heating. Energy Convers. Manag..

[62] Lu C., Gao L., Zhang L., Liu K., Hou Y., He T., Zhou Y., Wei G. (2022). Selective catalytic transfer hydrogenation of polyunsaturated fatty acid methyl esters using Pd/organobentonite as catalyst under microwave heating. Chem. Eng. Process..

